# MiR-103a-3p promotes tumour glycolysis in colorectal cancer via hippo/YAP1/HIF1A axis

**DOI:** 10.1186/s13046-020-01705-9

**Published:** 2020-11-20

**Authors:** Zhenqiang Sun, Qiuge Zhang, Weitang Yuan, Xiaoli Li, Chen Chen, Yaxin Guo, Bo Shao, Qin Dang, Quanbo Zhou, Qisan Wang, Guixian Wang, Jinbo Liu, Quancheng Kan

**Affiliations:** 1 Department of Colorectal Surgery, The First Affiliated Hospital, Zhengzhou University, Zhengzhou, 450052 Henan China; 2 Academy of Medical Sciences, Zhengzhou University, Zhengzhou, 450052 Henan China; 3 Department of Geriatric Medicine, The First Affiliated Hospital, Zhengzhou University, Zhengzhou, 450052 Henan China; 4 School of Life Science, Zhengzhou University, Zhengzhou, 450001 Henan China; 5 School of Basic Medical Sciences, Zhengzhou University, Zhengzhou, 450002 Henan China; 6 Henan Academy of Medical and Pharmaceutical Sciences, Zhengzhou University, Zhengzhou, 450052 Henan China; 7 Department of Gastrointestinal Surgery, The Affiliated Tumor Hospital, Xinjiang Medical University, Xinjiang, 830000 Urumqi China; 8 Department of Pharmacy, The First Affiliated Hospital, Zhengzhou University, Zhengzhou, 450052 Henan China

**Keywords:** MiR-103a-3p, Hippo pathway, Glycolysis, Colorectal cancer

## Abstract

**Background:**

Glycolysis plays an essential role in the growth and metastasis of solid cancer and has received increasing attention in recent years. However, the complex regulatory mechanisms of tumour glycolysis remain elusive. This study aimed to explore the molecular effect and mechanism of the noncoding RNA miR-103a-3p on glycolysis in colorectal cancer (CRC).

**Methods:**

We explored the effects of miR-103a-3p on glycolysis and the biological functions of CRC cells in vitro and in vivo. Furthermore, we investigated whether miR-103a-3p regulates HIF1A expression through the Hippo/YAP1 pathway, and evaluated the role of the miR-103a-3p-LATS2/SAV1-YAP1-HIF1A axis in promoting glycolysis and angiogenesis in CRC cells and contributed to invasion and metastasis of CRC cells.

**Results:**

We found that miR-103a-3p was highly expressed in CRC tissues and cell lines compared with matched controls and the high expression of miR-103a-3p was associated with poor patient prognosis. Under hypoxic conditions, a high level of miR-103a-3p promoted the proliferation, invasion, migration, angiogenesis and glycolysis of CRC cells. Moreover, miR-103a-3p knockdown inhibited the growth, proliferation, and glycolysis of CRC cells and promoted the Hippo-YAP1 signalling pathway in nude mice in a xenograft model. Here, we demonstrated that miR-103a-3p could directly target LATS2 and SAV1. Subsequently, we verified that TEAD1, a transcriptional coactivator of Yes-associated protein 1 (YAP1), directly bound to the HIF1A promoter region and the YAP1 and TEAD1 proteins co-regulated the expression of HIF1A, thus promoting tumour glycolysis.

**Conclusions:**

MiR-103a-3p, which is highly expressed in CRC cells, promotes HIF1A expression by targeting the core molecules LATS2 and SAV1 of the Hippo/YAP1 pathway, contributing to enhanced proliferation, invasion, migration, glycolysis and angiogenesis in CRC. Our study revealed the functional mechanisms of miR-103a-3p/YAP1/HIF1A axis in CRC glycolysis, which would provide potential intervention targets for molecular targeted therapy of CRC.

## Background

Colorectal cancer (CRC) is one of the leading causes of cancer-related deaths worldwide [1–3]. Due to the strong proliferation, invasion and metastatic ability of tumour cells, high energy metabolism is needed to provide the required energy. As the tumour volume increases, an anoxic state will inevitably occur in the tumour. Therefore, tumour cells have their own specific mechanism of cell metabolism, that is, a metabolic mode mainly based on glycolysis [4–6]. If the glycolytic pathway of CRC cells is effectively inhibited, tumour progression can be controlled. However, at present, there is still no obvious effective drug targeting tumour cell metabolism. Therefore, studying the specific mechanism of CRC metabolism and finding effective molecular diagnostic and treatment targets is expected to prolong the survival of patients and improve prognosis.

Research on the mechanism of tumour metabolic regulation is still in the preliminary exploration stage. In the hypoxic tumour microenvironment, hypoxia stimulates high expression of hypoxia-inducible factor (HIF) in cancer cells [7]. HIF inhibits the oxidative phosphorylation pathway in cancer cells and further enhances the glycolytic pathway. This metabolic phenomenon is known as the Warburg effect [8–10]. In a variety of malignant tumours, RAS [11], PI3K/Akt [12], BCR-ABL [13] and other oncogenes all promote glycolysis and decrease mitochondrial oxidative phosphorylation. Our previous findings reported that YAP1, as an oncogene, was involved in CRC progression [14, 15]. YAP1 was reported to promote glycolysis in cancer [16, 17].

The Hippo pathway is a newly discovered evolutionarily conserved inhibitory signalling pathway [18]. The Hippo pathway has important regulatory effects on organ size, tumourigenesis, tumour metabolism, stem cell homeostasis, and mesenchymal transition [19, 20]. In mammals, central to this pathway is a kinase cascade that includes the MST1, MOB1, LATS1, LATS2 and SAV1 kinases [21, 22]. One of the major targets of the Hippo core kinase cascade is YAP, which is phosphorylated and inhibited by activated LATS2 and SAV1 [23–25]. However, once the Hippo pathway is inactivated, non-phosphorylated YAP translocates into the nucleus, interacts with the transcription factor TEADs and then drives target gene expression [19, 24]. In recent years, research reports on the regulation of the Hippo pathway by miRNAs have gradually attracted attention. For example, it has been demonstrated that miR-135b regulates the Hippo pathway to promote lung cancer metastasis [26], and miR-31 inhibits the expression of LATS2 via the Hippo pathway and promotes epithelial-mesenchymal transition in esophageal squamous cell carcinoma (ESCC) [27]. At present, the research of miRNAs involved in the Hippo pathway on the regulation of metabolism is still in the primary exploration stage. Therefore, research on the function and mechanism of miRNAs affecting tumour metabolism via the Hippo pathway will open up a new field for the diagnosis and treatment of tumours.

The development of CRC is a multi-step process involving multiple factors, including altered expression levels of various non-coding RNAs (ncRNAs) [28]. Increasing evidence suggests that abnormal expression of miRNAs is involved in oncogenesis, proliferation, metastasis and invasion of CRC. Among these miRNAs, studies have shown that miR-103a-3p is an oncogene and is upregulated in hepatocellular carcinoma [29], endometrial carcinoma [30] and gastric cancer [31]. In addition, miR-103a-3p was shown to promote the occurrence, proliferation, and metastasis of CRC and was associated with poor prognosis in patients with CRC, meanwhile, miR-103a-3p was reported to target LATS2, ZO1, et al. [32–34]. However, the molecular mechanism by which miR-103a-3p regulates tumour metabolism via the Hippo pathway and promotes CRC invasion and metastasis remains largely unknown.

We firstly proposed the hypothesis that miR-103a-3p may regulate CRC glycolysis through the Hippo pathway to promote the invasion and metastasis of CRC and then explored it. In this study, the expression of miR-103a-3p in CRC cells and tissues was confirmed to increase. MiR-103a-3p promotes YAP1/TEAD-mediated expression of HIF1A by directly targeting LATS2 and SAV1 and further promotes CRC glycolysis.

## Materials and methods

### Patient tissue specimens

Forty paired CRC tissues and matched adjacent nontumour tissues were obtained from patients after receiving surgical resection at The First Affiliated Hospital of Zhengzhou University. None of the patients received any preoperative chemotherapy or radiotherapy. Survival was calculated by months. Overall survival (OS) was defined as the time from tumour excision to death by any cause. Pathological diagnoses of colorectal cancer were determined by three pathologists. The tumour stage was determined according to the eighth edition of the International Union Against Cancer (UICC)/American Joint Committee on Cancer (AJCC) TNM classification [35]. All patients signed informed consent forms, and this protocol was approved by the Ethics Committee of The First Affiliated Hospital of Zhengzhou University.

### Cell culture, transfection and stable cell line construction

HCT116 cells were obtained from iCell Bioscience Inc. (Shanghai, China) and authenticated by STR before use. SW480 cells were obtained from the Biotherapy Centre of The First Affiliated Hospital of Zhengzhou University. All cells were cultured in DMEM (high glucose) (HyClone, Logan, Australia) with 10% foetal bovine serum (BI, Israel), 100 U/ml penicillin, and 100 mg/ml streptomycin at 37 °C and 5% CO2. A plasmid containing the precursor sequence of miR-103a-3p (pre-miR-103a-3p) was obtained from Vigene (Rockville, MD). The pMIF-cGFP-ZEO/miR-103a-3p plasmid, pSilencer-1-cGFP/miR-103a-3p shRNA plasmid were obtained from RiboBio (Guangzhou, China). The pLVX-TRE3G-ZsGreen1/YAP1, pLVX-TRE3G-ZsGreen1/HIF1A and pLVX-TRE3G-ZsGreen1/TEAD1 plasmids were purchased from Vigene, and the corresponding lentiviruses/shRNAs were purchased from Vigene. The psiCHECK-2-LATS2–3′-UTR and psiCHECK-2-SAV1–3′-UTR WT plasmids and the corresponding mutants were purchased from Vigene. According to the manufacturer’s instructions, Lipofectamine 3000 (Invitrogen, ThermoFisher Scientific, Carlsbad, CA, USA) was used for transient siRNA and plasmid transfection. LATS2, SAV1, HIF1A, YAP1, and TEAD1 siRNAs were purchased from RiboBio. The siRNA sequences are shown in Table S1. MiR-103a-3p mimics and inhibitors were stably transfected into SW480 cells and HCT116 cells, respectively. Then, the cells were cultured with puromycin to obtain stable cell lines.

### RNA extraction and quantitative real-time PCR

Total RNA was extracted from cells and tissues with RNAiso Plus reagent (Takara, Dalian, China) according to the manufacturer’s instructions. The concentration and purity of RNA were detected using a NanoDrop 2000 (Thermo Scientific, USA). First-strand cDNA was synthesized from 1 μg of total RNA using the Prime Script RT Master Mix Kit (Takara), and real-time PCR was performed using GoTaq qPCR Master Mix (Vazyme, Nanjing, China) according to the manufacturer’s instructions. 2^-ΔΔCt^ method was used to calculate relative levels of genes and miRNA expression. The primers are listed in Table S1. GAPDH or U6 was used as an endogenous control for normalization. The relative expression was calculated.

### Western blotting

Total proteins were extracted by RIPA buffer supplemented with PMSF (Solarbio, Beijing, China) and quantified by a BCA kit. Then, the proteins were separated in SDS-PAGE gels and transferred into PVDF membranes (Millipore, Massachusetts, USA). Membranes were blocked with TBST with 5% skim milk powder and incubated overnight at 4 °C with primary antibodies against YAP1 (1:1000), P-YAP1 (1:1000), VEGFA (1:1000), Hexokinase II (HK2; 1:5000), LDHA (1:2000), HIF1A (1:1000), GAPDH (1:5000), Tublin (1:1000), and β-actin (1:1000) (Proteintech, Wuhan, China). The next day, blots were washed with PBS and then secondary antibodies were incubated with the membrane at room temperature for 1 h. The membrane was visualized using a chemiluminescence kit (Absin, Shanghai, China) and quantified by densitometry analysis using ImageJ software. GAPDH and tubulin were used as loading controls.

### Wound-healing assay

The transfected HCT116 and SW480 cells were seeded in 12-well plates. After the cells were grown to 80–90% confluence, scratch wounds were generated by a 10 μl plastic pipette tip, which was recorded as 0 h. Cell migration was assessed by measuring the movement of cells into the scratch wounds. Then, the scratch was imaged at 24 h, 48 h, 72 h, and 96 h. Wound width was measured with an ocular ruler to ensure that all wounds were the same width at the beginning of each experiment.

### Transwell assays

To assess the migration and invasiveness of HCT116 and SW480 cells, we used Transwell chambers (Corning, NY, USA). Briefly, approximately 3 × 10^5^ cells in serum-free medium were seeded in the upper chambers with 8 μm pore size membranes to perform the migration assay in the absence of Matrigel (Corning, NY, USA) and the invasion assay with Matrigel. Dulbecco’s modified Eagle’s medium (500 μl) supplemented with 10% foetal bovine serum was added to the lower chamber. After incubation in a humidified atmosphere containing 5% CO_2_ at 37 °C for 72 h, the migrated cells were fixed, and the other cells were wiped off. Then, the migrated cells were stained by Giemsa (Solarbio, Beijing, China). Stained cells were imaged under an IX53 inverted microscope (Nikon, Tokyo, Japan), and the Image-Pro Plus software programme (Media Cybernetics, Rockville, MD) was used to count the cells.

### Cell proliferation assay

A total of 2 × 10^3^ cells per well were seeded into 96-well plates. Cell proliferation was evaluated using Cell Counting Kit-8 (Dojin Laboratories, Tokyo, Japan) according to the manufacturer’s instructions. We collected cell samples at 24 h, 48 h, 72 h, 96 h, and 120 h. Then, 10 μl of CCK-8 solution was added to the culture medium and incubated for 2 h at 37 °C. Viable cells were evaluated by measuring the absorbance at 450 nm with a reference wavelength of 570 nm.

### 5-Ethynyl-2′-deoxyuridine assay (EdU)

A total of 2 × 10^3^ cells per well were seeded into 96-well plates, cultured overnight, washed with phosphate-buffered saline (PBS), fixed with 4% paraformaldehyde for 30 min, and incubated with 2 mg/ml glycine for 5 min. Based on the kFluor488-EdU manufacturer’s instructions (RiboBio), 200 μl of 1× Apollo dyeing solution was added to each well, followed by incubation at room temperature for 30 min. Next, 100 μl of 0.5% Triton X-100 was used to wash the cells two to three times (10 min per wash). Following staining with Hoechst 33342 at room temperature for 30 min in darkness and one or two washes with PBS, the cells were observed using a Micro imaging system (ImageXpress, Downingtown, PA, USA). Five fields were randomly selected and imaged, and the number of EdU-positive cells was calculated.

### Tube formation assay

Twenty-four-well plates were coated with 60 ml Matrigel (BD Biosciences, USA) at 37 °C for 1 h for gel formation. 1 × 10^5^ HUVEC were co-incubated with supernatants from each group stably transfected cells for 10 min in the pre-solidified Matrigel and allowed to start the process of forming capillary tubes and networks once seeded on Matrigel. Six hours after incubation, the plates were observed under a microscope and imaged (Nikon, Japan). The numbers of branching points generating at least three tubules were counted.

### Dual-luciferase reporter assay

Luciferase activity assays were performed with the Dual-Luciferase Reporter Assay System (Promega, Beijing, China). Validation of miRNA targets was performed by cloning partial LATS2 (SAV1) 3′-UTRs containing the sequence recognized by the miR-103a-3p seed sequence. HCT116 cells and HEK293 T cells were cotransfected with miR-103a-3p mimics, the *Renilla* luciferase reporter vector (Promega), and either wild-type (WT) or mutant LATS2 (SAV1) reporter constructs using Lipofectamine 2000 reagent (Life Technologies) according to the manufacturer’s instructions. After 48 h of transfection, firefly and *Renilla* luciferase activities were measured using the Dual-Luciferase Reporter Assay System (Promega). Correction for differences in transfection efficiency was performed by normalizing firefly luciferase activity to total *Renilla* luciferase activity.

### Immunofluorescence assay

HCT116 cells transfected with miR-103a-3p inhibitors were fixed by 4% paraformaldehyde and permeabilized by 0.1% Triton X-100 in PBS for 10 min. The cells were blocked with 5% BSA for 60 min at room temperature and incubated with primary antibody against YAP1 (1:100) and HIF1A (1:500) overnight at 4 °C. The next day, the cells were washed with PBS (0.1% Triton X-100) three times and then incubated with HRP conjugated secondary antibody for 60 min at room temperature, followed by nuclear staining with DAPI. Fluorescent images were acquired using an OLYMPUS FV1000 confocal microscope.

### Chromatin immunoprecipitation (ChIP)

ChIP assays were performed as described previously [15]. Briefly, HCT116 cells were fixed in 1% formaldehyde for 10 min at room temperature. Then the formaldehyde-fixed cells were collected, lysed and sonicated for 10 cycles of 30 s on and 10 s off to obtain chromatin fragments. 10 μg chromatin fragments were incubated with 10 μl antibodies against YAP1(1:50), 10 μl antibodies against TEAD1 (1:100) or 2 μl antibodies against rabbit IgG (1:100) at 4 °C overnight. The next day, the generated immuno-complexes were washed with lysis buffer (0.1 M NaHCO3, 1% SDS) four times and then decrosslinking was carried out for 4 h at 65 °C. The associated genomic DNA was separated by SDS-PAGE. The precipitated DNA was subjected to PCR amplification. PCR was performed with HIF1A promoter-specific primers that amplified the YAP1/TEAD1 binding regions. The primers were HIF1A Forward 5′-TACTCAGCACTTTTAGATGCTGTT-3′ and Reverse: 5′-ACGTTCAGAACTTATCCTACCAT-3′.

### Measure the PH value in cell nutrient solution

The PH value of cell nutrient solution was measured by pH Meter. The pH meter measured results in increments of 0.1 pH units, between 4.0 and 8.0. According to the manufacturer’s instructions, before analysis, the pH Meter needed to be calibrated with pH 6.8 buffer solution. PH detection method is to insert the glass electrode into the cell culture medium to be measured, and then put another electrode. Between measurements, the pH sensor and container were rinsed with pure water.

### Seahorse assay

The Seahorse XF 96 Extracellular Flux Analyzer (Agilent) was used to determine the extracellular acidification rate (ECAR). According to the manufacturer’s instructions, ECAR was examined with a Seahorse XF glycolysis stress test kit. Briefly, 2 × 10^4^ HCT116 or SW480 cells per well with different treatments were seeded into a Seahorse XF 96 cell culture plate with 15% fetal bovine serum DMEM overnight. Cells were washed and incubated with base medium with 2 mML-glutamine for 1 h at 37 °C, CO2-free incubator. After 3 baseline measurements, glucose, oligomycin, and 2-DG was sequentially added d into each well at the time points specified to a final concentration of 10 mM, 10 μM or 50 mM, respectively. ECAR dates were assessed by Seahorse XF 96 Wave software.

### Subcutaneous xenotransplantation model

All mouse procedures were approved by the Institutional Animal Care and Use Committee of Zhengzhou University. All BALB/c nude mice, 6 weeks old, were acquired from Vital River Laboratory (Beijing, China). Logarithmic phase HCT116 cells (1 × 10^6^/100 μl) were inoculated subcutaneously into the dorsal flank. After 12 days, according to the completely randomized design using a random comparison table, the mice with xenograft tumours were randomly divided into two groups, intratumoural injection of miR-103a-3p antagomir group and negative control group, to examine tumourigenicity. These mice were treated with miR-103a-3p antagomir every 3 days. The tumour size was measured by a slide calliper, and tumour volume was evaluated by the following formula: volume = (D × d^2^)/2, where D was the longest diameter and d was the shortest diameter. All animals were sacrificed 32 days after inoculation, and the tumours were excised, weighed, fixed, and paraffin embedded for haematoxylin-eosin (H&E) and immunohistochemistry (IHC) staining detected under a microscope. All specimens were examined under a light microscope (Nikon, Japan).

### Immunohistochemistry

Immunohistochemical staining was performed as previously described [36]. Xenograft tumours for H&E and IHC were fixed in formalin, embedded in paraffin, and sectioned at 4-mm thickness after embedding. The deparaffinized sections were performed antigen retrieval in the boiling citrate buffer (0.01 M, pH 6.0) for 10 min. The sections were performed with antibodies against the following antigens (Cell Signalling Technology): P-YAP (1:1000), HK2 (1:5000), PKM1 (1:2000), PCNA (1:1000), and KI-67 (1:500) overnight at 4 °C. The next day, the sections were incubated with the anti-goat IgG-HRP (Santa Cruz Biotechnology) secondary antibody at 37 °C for 30 min. For immunohistochemistry, immunodetection was performed using DAB as the chromogen, and hematoxylin as nuclei counter-stain. Laser scanning confocal microscopy was used to capture images of the tumours.

### Statistical analysis

All statistical analyses were carried out with SPSS version 18.0 (MT, USA) and GraphPad Prism 5.0 software (CA, USA). Data are expressed as the mean ± SEM. All differences between two independent groups were evaluated by a two-tailed Student’s *t*-test. Survival curves were generated using the Kaplan–Meier method and compared using the log-rank test. The MedCalc software was used to generate the ROC curve, and the data were analyzed by two-tailed t test. Pearson’s coefficient was used to assess the correlation between two independent groups. The associations of miR-103a-3p expression and clinicopathologic variables were assessed by the Chi-square test or Fisher’s exact test. The indicated *P* values (*P < 0.05 and **P < 0.01) were considered statistically significant.

## Results

### MiR-103a-3p is an oncogene in CRC and is correlated with poor prognosis in CRC patients

**Fig. 1:**
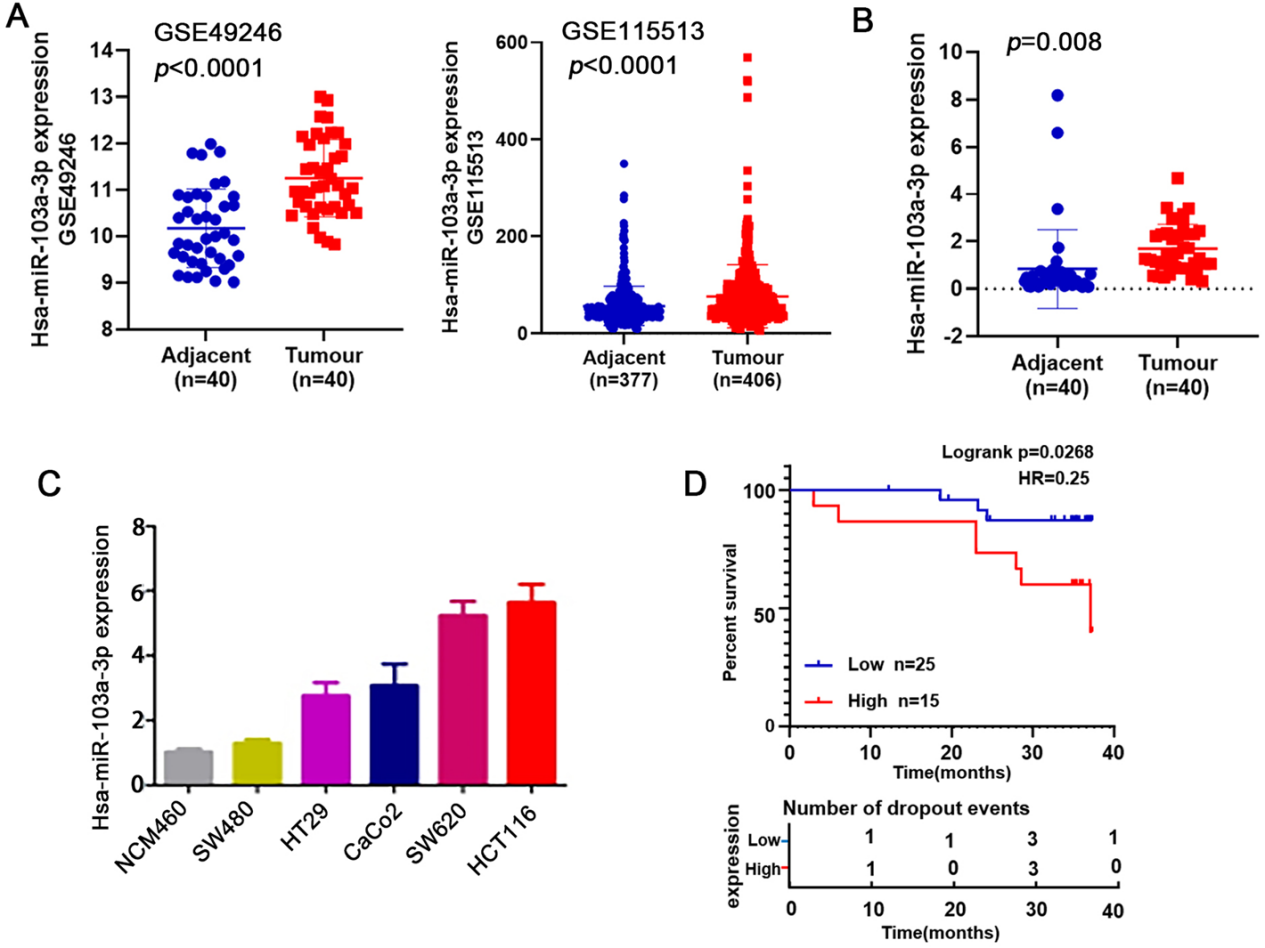
Aberrant expression of miR-103a-3p in CRC tissues and cell lines. **a** The GSE49246 and GSE 115513 datasets showing upregulation of miR-103a-3p in CRC. **b** qRT-PCR assay showing an upregulation of miR-103a-3p in 40 paired CRC tissues and matched normal tissues. **c** qRT-PCR assay showing a general upregulation of miR-103a-3p in different CRC cell lines. **d** Kaplan-Meier analysis showing the level of miR-103a-3p is negatively correlated with overall survival rate of CRC patients

To assess the role of miR-103a-3p in CRC, we first quantitated miR-103a-3p gene expression levels in CRC tissues and adjacent tissues using the microarray datasets GSE49246 and GSE115513 (*P* < 0.001; Fig. 1a). Using qRT-PCR, it was confirmed that miR-103a-3p was highly expressed in 40 paired CRC tissues and adjacent normal tissues (Fig. 1b). Moreover, among the cell lines tested, the expression level of miR-103a-3p was the highest in HCT116 cells, and the expression level of miR-103a-3p was the lowest in SW480 cells (NCM460 is a normal colon mucosal cell line) (Fig. 1c). In a cohort of 40 CRC cases, the patients were divided into low and high expression groups of miR-103a-3p. Univariate analysis showed that the high expression of miR-103a-3p was associated with distant metastasis (Tables S1). Interestingly, patients with high miR-103a-3p levels had a worse prognosis and shorter survival time than those with low miR-103a-3p expression (log-rank test, *p* < 0.05; Fig. 1d). These findings suggested that miR-103a-3p may play an oncogenic role in CRC.

### In a hypoxic environment, miR-103a-3p promotes CRC cell proliferation, invasion, migration, angiogenesis and glycolysis in vitro

**Fig. 2:**
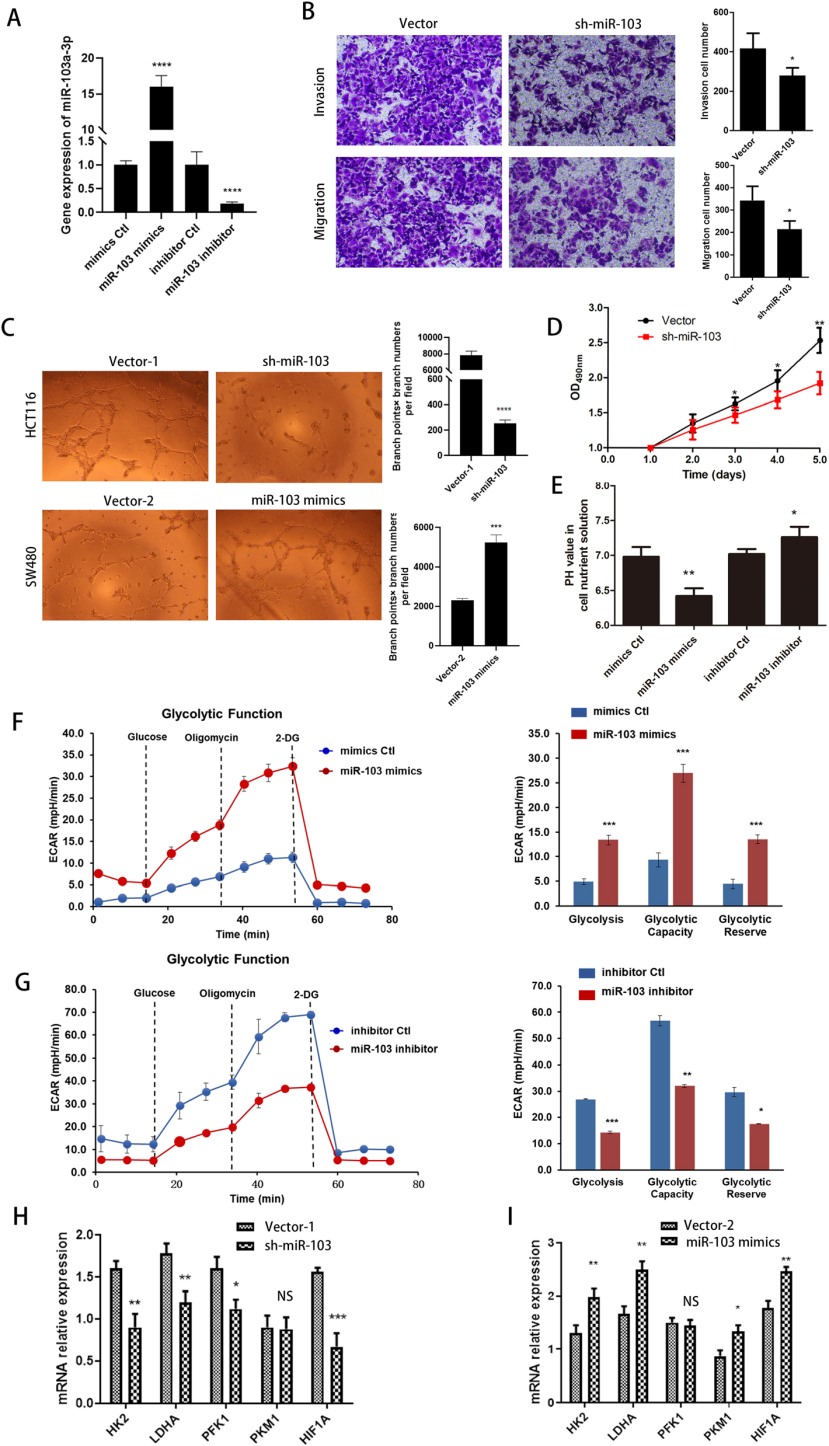
MiR-103a-3p serve as oncogenic roles and promote glycolysis in CRC cells. **a** qRT-PCR assay indicating the levels of miR-103a-3p in SW480 and HCT116 cells stably transfected with miR-103a-3p mimics, mimics control, miR-103a-3p inhibitor or inhibitor control. **b** Transwell and **d** CCK-8 assays indicating the invasion, migration and proliferation ability of HCT116 cells transfected with empty vector or miR-103a-3p shRNA (sh-miR-103a-3p). **c** Angiogenesis ability in HCT116 and SW480 cells transfected with vector-1, sh-miR-103a-3p, vector-2, or miR-103a-3p mimics. **e** The acidity of the cell culture medium was detected in HCT116 and SW480 cells transfected with vector-1, sh-miR-103a-3p, vector-2, or miR-103a-3p mimics under hypoxia. **f-g** The ECAR and the variations of glycolysis, glycolysis capacity and glycolysis reserve of CRC cells were analyzed by Seahorse XFe 96 Extracellular Flux Analyzer. **h-i** qRT-PCR assay showing the expression levels of *HK2*, *LDHA*, *PFK1*, *PKM1* and *HIF1A* in HCT116 and SW480 cells transfected with vector-1, sh-miR-103a-3p, vector-2, or miR-103a-3p mimics. *p < 0.05, **p < 0.01, ***p < 0.01

To investigate the physiological function of miR-103a-3p in CRC cells, the miR-103a-3p silencing and overexpression constructs were stably transfected into HCT116 and SW480 cells, respectively. The efficiency of overexpression and inhibition of miR-103a-3p was verified in colon cancer cells by qRT-PCR (Fig. 2a). Transwell and CCK-8 assays showed that miR-103a-3p silencing inhibited the invasiveness, metastasis and proliferation of HCT116 cells compared with the control group (Fig. 2b, d). In addition, knockdown or overexpression of miR-103a-3p reduced or increased the angiogenesis of HCT116 and SW480 cells, respectively, compared with those stably transfected with the corresponding empty vector (Fig. 2c). The acidity of the cell nutrient solution (i.e., the pH value) was significantly increased or decreased in SW480 or HCT116 cells with stable overexpression or knockdown of miR-103a-3p, respectively (Fig. 2e). Subsequently, we conducted the effect of miR-103a-3p overexpression and knockdown on glycolytic metabolism via seahorse assay. The result shows that the overexpression miR-103a-3p enhanced the extracellular acidification rate (ECAR), indicated that HCT116 cells transfected with miR-103a-3p mimics produced more extracellular lactate and enhanced the glycolytic metabolism compared with those transfected with mimics control (Fig. 2f). Knockdown of miR-103a-3p suppressed the ECAR of SW480 cells (Fig. 2g). Moreover, analysis using the TCGA dataset demonstrated positive associations between miR-103a-3p and *HK2* and *HIF1A* (Fig. S1A, Supporting Information). We next examined the effect of miR-103a-3p on key molecules of glycolysis and on *HIF1A* by qRT-PCR. The results demonstrated that stable knockdown of miR-103a-3p decreased the transcript levels of *HIF1A* and its downstream glycolytic genes *HK2*, *LDHA*, and *PFK1* in HCT116 cells, while the overexpression of miR-103a-3p increased the transcript levels of *HIF1A* and its downstream glycolytic genes *HK2*, *LDHA*, and *PKM1* in SW480 cells compared with those in cells stably transfected with empty vector (Fig. 2h, i). Furthermore, we analysed the prognostic value, sensitivity and specificity of glycolytic genes in CRC patients from TCGA database. We found that higher expression of PFK1 or PKM1 was associated with lower survival probability (Fig. S1B, Supporting Information). The area under the ROC curve was used to determine the diagnostic value of glycolytic genes for CRC. The AUC of HIF1A, PKM1, LDHA, PFK1 and HK2 was 0.575, 0.928, 0.713, 0.839 and 0.713, respectively (Fig. S1C, Supporting Information). These results revealed that miR-103a-3p promotes tumour progression and glycolysis in CRC.

### Knockdown of miR-103a-3p suppresses CRC growth, proliferation, angiogenesis, and glycolysis in vivo

**Fig. 3:**
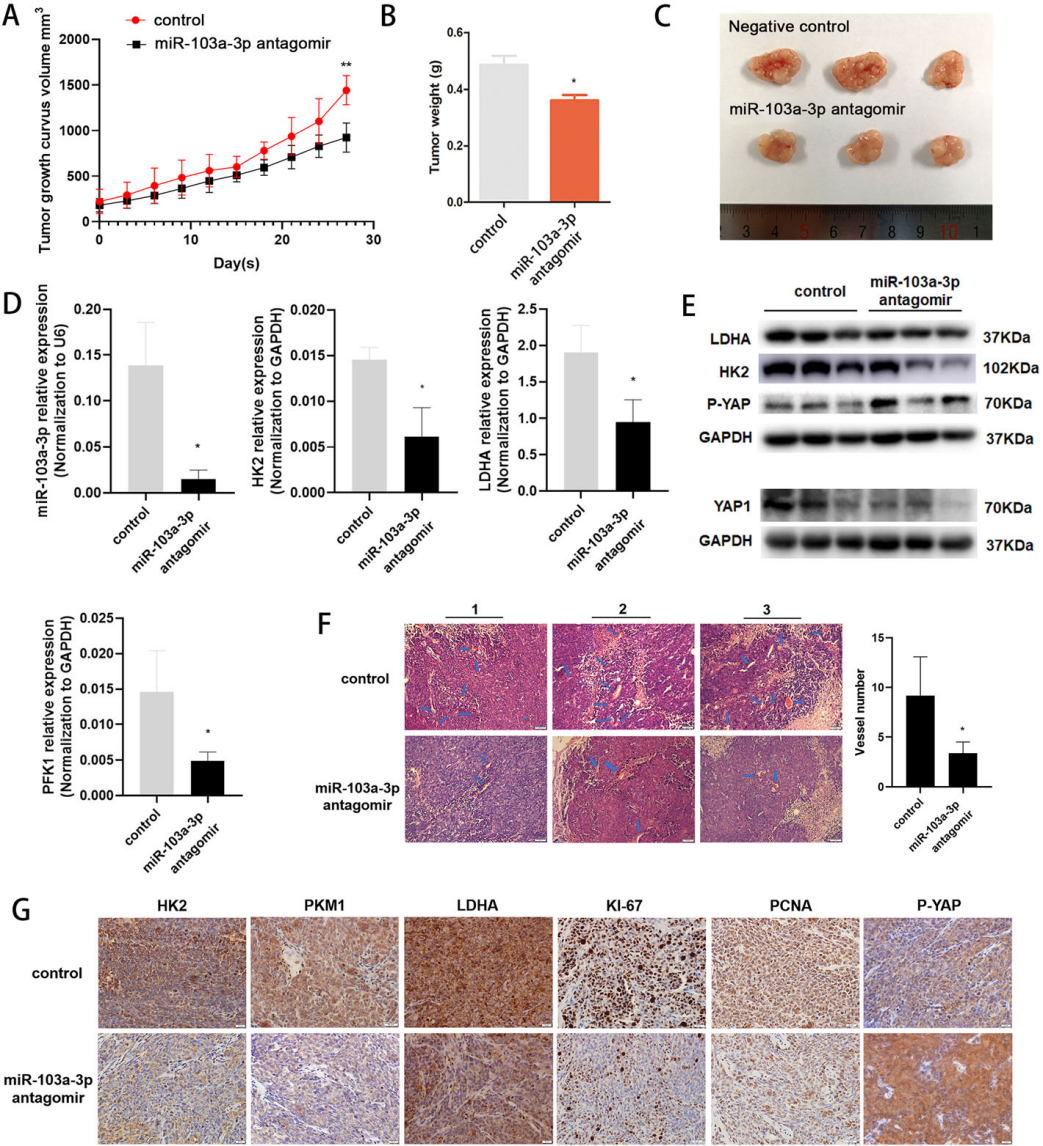
MiR-103a-3p knockdown suppresses the growth, proliferation, angiogenesis, and glycolysis of CRC in vivo. Nude mice were subcutaneous injected with HCT116 cells transfected with antagomir control and miR-103a-3p antagomir. **a** MiR-103a-3p knockdown inhibited tumour growth. Tumour volume was measured every 3 d using the formula: volume = (length × width^2^)/2. **b** The tumours weight was measured after dissecting from the nude mice at 37 d after injection. **c** Representative image of xenograft tumours was shown. **d** The level of miR-103a-3p, *HK2*, *LDHA*, and *PFK1* were determined in tumour tissues from miR-103a-3p antagomir group and control group by qRT-PCR. **e** Western blot assays revealing the protein levels of LDHA, HK2, P-YAP and YAP1 in tumour tissues from miR-103a-3p antagomir group and control group. **f** Representative images showing vascular distribution and density within tumour tissues from miR-103a-3p antagomir group and control group by H&E staining (scale, 50 μm). **g** Immunohistochemical staining showing the protein levels of HK2, PKM1, LDHA, KI-67, PCNA, and P-YAP in tumour tissues from miR-103a-3p antagomir group and control group (scale, 50 μm). *p < 0.05, **p < 0.01, ***p < 0.001

To further confirm the in vitro findings, we observed the biological roles of miR-103a-3p in vivo. We injected HCT116 cells subcutaneously into the dorsal flanks of athymic nude mice to establish xenograft tumour model. One week later, the mice were randomly divided into an intratumoural injection of miR-103a-3p antagomir group and antagomir control group. The tumours were extracted after 3 weeks of drug treatment. Our results showed that the growth and weight of xenograft tumours treated with miR-103a-3p antagomir were lower than those of xenograft tumours treated with antagomir control (Fig. 3a-c). In addition, the expression levels of miR-103a-3p and the key glycolytic molecules *HK2*, *LDHA* and *PFK1* in tumour tissues from miR-103a-3p antagomir group were lower than control values (Fig. 3d). Consistent with the above results, miR-103a-3p knockdown downregulated the protein expression of HK2 and LDHA. In addition, Western blot assays showed that the inhibition of miR-103a-3p decreased YAP1 expression and increased P-YAP expression, respectively (Fig. 3e). Furthermore, we observed the angiogenic ability of the two groups of tumour tissues by H&E staining. The results indicated that miR-103a-3p knockdown could inhibit tumour angiogenesis (Fig. 3f). Immunohistochemical staining revealed that the protein expression of key glycolytic molecules HK2, LDHA, and PFK1 and the proliferation-related nuclear factors KI-67 and PCNA were downregulated in miR-103a-3p antagomir group, while the protein expression of P-YAP was significantly increased (Fig. 3g). TCGA CRC database revealed that miR-103a-3p expression positively correlated with YAP1 levels in the CRC tissues (Fig. S1D, Supporting Information). Collectively, our results suggest that miR-103a-3p silencing can restrict CRC cell growth, proliferation, glycolysis and angiogenesis in vivo. Based on overall evidences in the study, we speculate that miR-103a-3p participate in the regulation of the Hippo-YAP signalling pathway.

### MiR-103a-3p directly targets the core molecules LATS2 and SAV1 of the hippo pathway

**Fig. 4:**
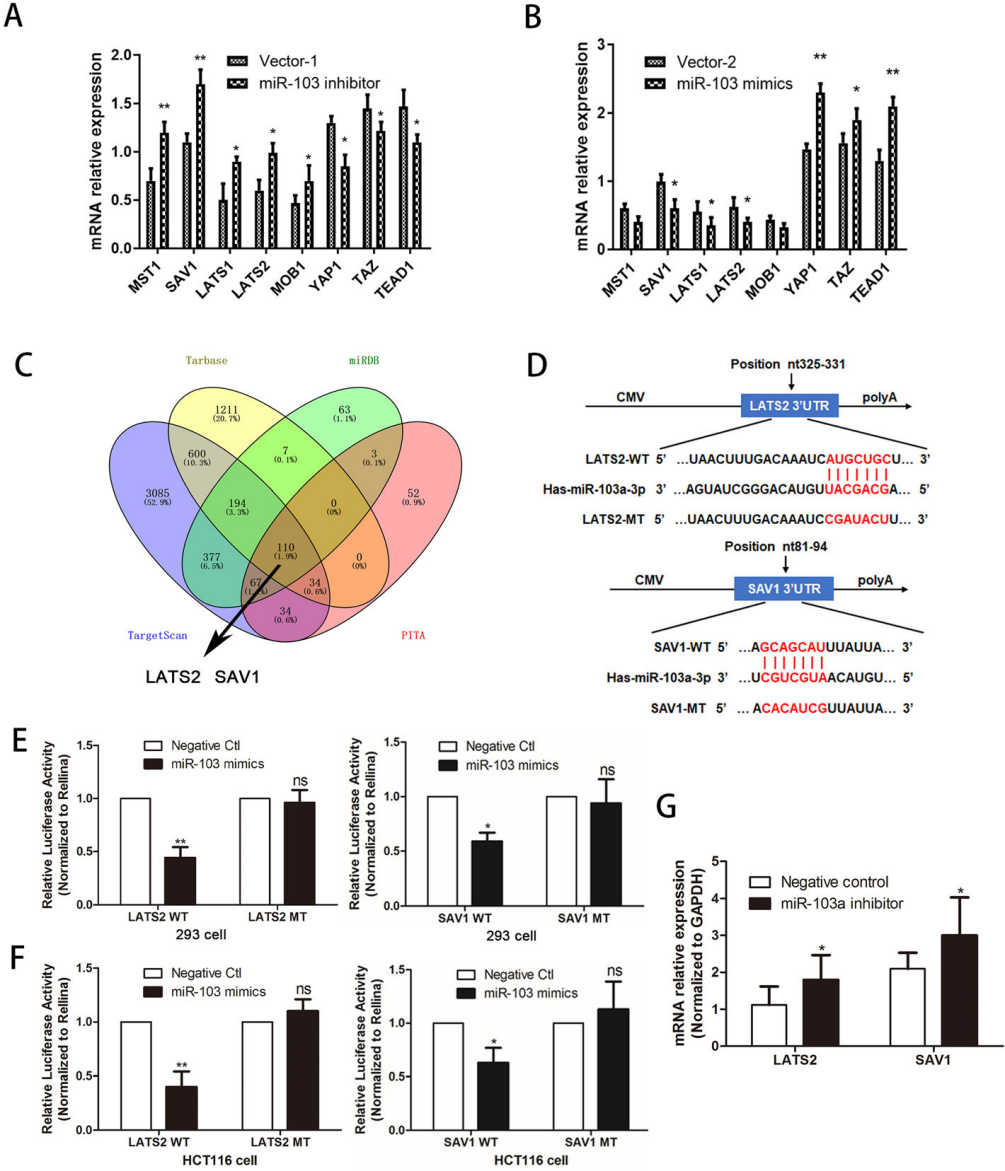
MiR-103a-3p inhibits the Hippo pathway by directly targeting LATS2 and SAV1. **a-b** The mRNA levels of the core molecules of the Hippo pathway were detected in HCT116 and SW480 cells transfected with vector-1, sh-miR-103a-3p, vector-2, or miR-103a-3p mimics. **c** Venn diagrams exhibiting the shared targets of miR-103a-3p predicted from the TargetScan, miRDB, Tarbase and PITA databases. **d** Scheme of the potential binding sites of miR-103a-3p in the LATS2/SAV1 3′UTR. Luciferase assay in 293 T cells **e** and HCT116 cells **f**. MiR-103a-3p mimics or mimic controls were cotransfected with pReporter-LATS2(SAV1)-WT (wild type) 3′UTR or pReporter-LATS2(SAV1)-MT (mutant type) 3′UTR. Luciferase activity was decreased in the pReporter-LATS2(SAV1)-WT group. **g** The levels of LATS2 and SAV1 were determined in tumour tissues from miR-103a-3p antagomir group and control group by qRT-PCR. *p < 0.05, **p < 0.01

To verify that miR-103a-3p is involved in the Hippo pathway, we examined the effect of miR-103a-3p on the core molecules of the Hippo pathway by qRT-PCR, such as a series of upstream kinases MST1, MOB1, LATS1, LATS2 and SAV1, downstream effectors YAP and TAZ, and transcription co-activator TEAD1. The results showed that stable knockdown of miR-103a-3p increased the transcript levels of *MST1, MOB1, LATS1, LATS2* and *SAV1* and decreased the transcript levels of *YAP1*, *TEAD1* and *TAZ* in HCT116 cells, while the overexpression of miR-103a-3p decreased the transcript levels of *LATS1, LATS2* and *SAV1* and increased the transcript levels of *YAP1*, *TEAD1* and *TAZ* in SW480 cells compared with those stably transfected with empty vector (Fig. 4a, b). Then, to further explore the potential molecular mechanism of miR-103a-3p participating in the Hippo-YAP pathway, we predicted that LATS2 and SAV1 may be targets of miR-103a-3p based on the TargetScan, miRDB, Tarbase, and PITA databases and as demonstrated by Venn diagrams (Fig. 4c). Subsequently, we identified potential binding sites for miR-103a-3p in the 3’UTR of LATS2 (SAV1) mRNA using TargetScan (Fig. 4d). To validate whether LATS2 and SAV1 were direct targets of miR-103a-3p, a dual-luciferase reporter system containing the wild-type or mutant 3′UTR of LATS2 (SAV1) was used. Cotransfecting miR-103a-3p mimics with the wild-type LATS2 (SAV1) vector induced a decrease in luciferase activity in 293 T and HCT116 cells, whereas miR-103a-3p mimic control cotransfected with the mutant LATS2 (SAV1) vector had no effect (Fig. 4e, f), suggesting that miR-103a-3p directly and specifically bound the predicted binding site in the 3′UTR of LATS2 (SAV1). In addition, in xenograft tumours, we also confirmed that the expression of LATS2 and SAV1 was upregulated in the group treated with miR-103a-3p antagomir compared with the control group (Fig. 4g). Taken together, our results revealed that LATS2 and SAV1 were direct targets of miR-103a-3p.

### YAP1/TEAD1 affects glycolysis and angiogenesis by promoting the transcription of HIF1A

**Fig. 5:**
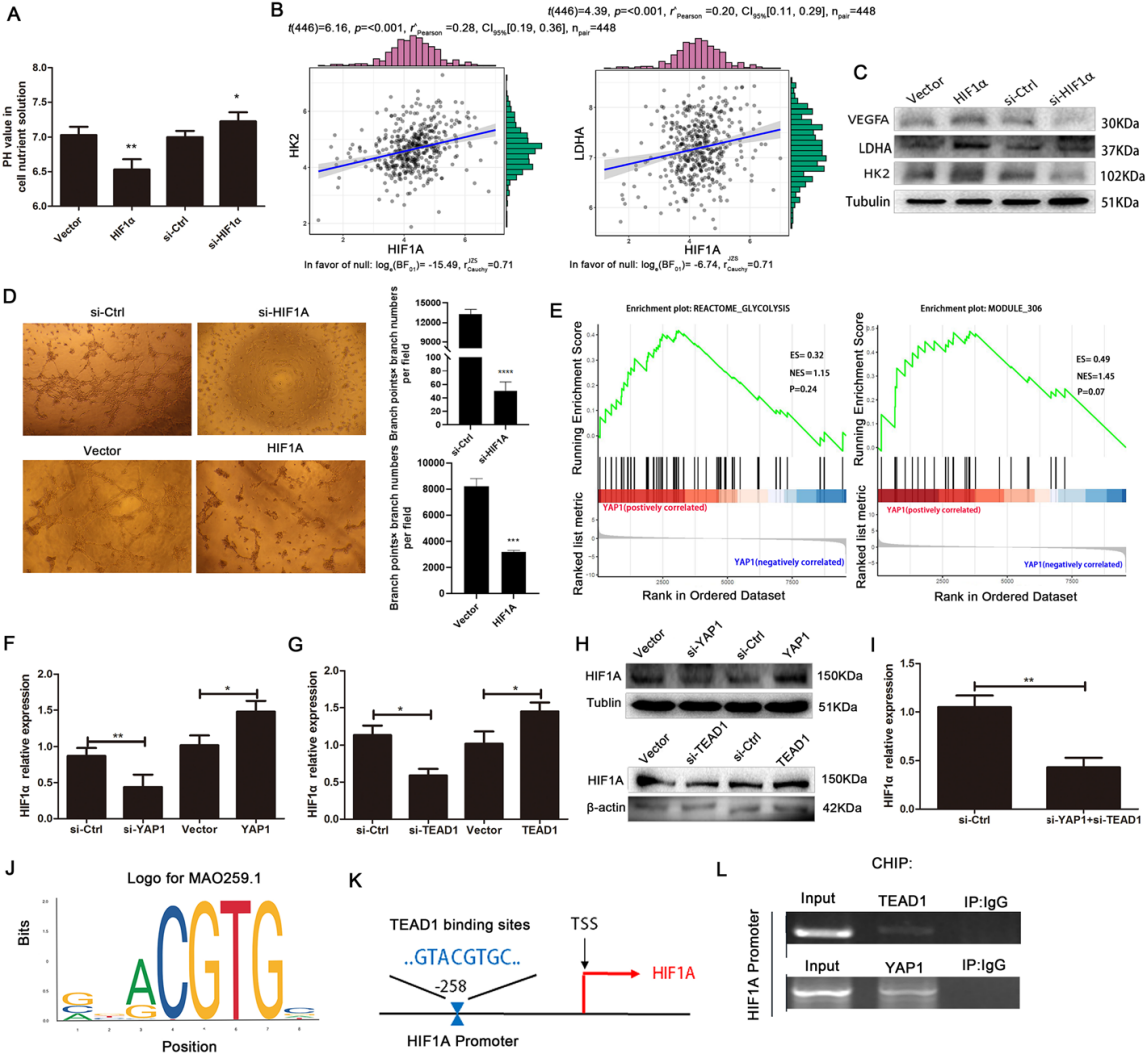
YAP1/TEAD1 promotes glycolysis and angiogenesis in CRC by activating the transcription of HIF1A. **a** The acidity of the cell nutrient solution was detected in HCT116 cells transfected with vector, HIF1A, si-control, or si-HIF1A under hypoxia. **b** The expression correlation of HIF1A with the glycolytic gene HK2 or LDHA in CRC samples by TCGA. **c** Western blot and angiogenesis **d** assays showing the expression of VEGFA, LDHA and HK2 and angiogenesis in HCT116 cells transfected with vector, HIF1A, si-control, or si-HIF1A under hypoxia. **e** Correlation analysis between YAP1 and glycolysis-associated gene sets, as demonstrated by GSEA. ES, enrichment score; NES, normalized enrichment score. The transcript **f-g** and protein levels **h** of HIF1A were measured in HCT116 cells transfected with vector, YAP1, TEAD1, si-YAP1, si-TEAD1, or si-control by qRT-PCR and western blot. **i** The level of HIF1A was determined in HCT116 cells transfected with si-YAP+si-TEAD1 or si-control by qRT-PCR. **j** The HIF1A binding motif in HIF1A predicted from JASPAR matrix models. **k** Scheme of the potential binding sites of TEAD1 in the HIF1A upstream promoter region. **l** ChIP assays using anti-YAP1 and anti-TEAD1 antibodies were performed in HCT116 cells. *p < 0.05, **p < 0.01

To investigate the role of HIF1A in CRC progression, a functional study of RNA overexpression and interference was performed. The acidity of the cell nutrient solution (i.e., the pH value) was significantly increased or decreased in HCT116 cells with stable overexpression or knockdown of HIF1A, respectively (Fig. 5a). We predicted that HIF1A levels were positively correlated with the glycolytic genes HK2 and LDHA in CRC using the TCGA dataset (Fig. 5b). Similarly, GEPIA database also revealed that HIF1A expression positively correlated with HK2 (Fig. S2A, Supporting Information) and LDHA (Fig. S2B, Supporting Information) in colon cancer, rectal cancer and CRC, respectively. Subsequently, we verified that the protein levels of VEGFA and the glycolytic genes HK2 and LDHA were increased or decreased in HCT116 cells with stable overexpression or knockdown of HIF1A, respectively (Fig. 5c). In addition, stable knockdown or overexpression of HIF1A reduced or increased the angiogenesis of HCT116 cells, respectively, compared with those stably transfected with empty vector (Fig. 5d). These results revealed that HIF1A promoted glycolysis and angiogenesis in CRC. Meanwhile, GSEA demonstrated positive associations between YAP1 and the gene sets “reactome glycolysis” and “module 306 (description: glycolysis and TCA cycle)” (Fig. 5e). We further investigated the interplay effects between YAP1 and TEAD1 in regulating HIF1A expression. TEAD1 is a well-known transcriptional coactivator of YAP1 [37]. Analysis using the GEPIA dataset indicates that the YAP1 levels were positively correlated with TEAD1 in colon cancer, rectal cancer and CRC (Fig. S3A, Supporting Information), and surprisingly, the YAP1 and TEAD1 levels were both positively correlated with HIF1A (Fig. S3B, Supporting Information) or LDHA (Fig. S3C, Supporting Information). Furthermore, HIF1A was positively regulated by YAP1 and TEAD1 in HCT116 cells as evaluated by qRT-PCR (Fig. 5f, g) and western blotting (Fig. 5h). Cotransfecting si-YAP1A with si-TEAD1 significantly decreased the transcript levels of HIF1A in HCT116 cells (Fig. 5i). Further bioinformatics analysis (JASPAR) showed that the HIF1A promoter region might have a DNA binding motif of TEAD1 (Fig. 5j, k), suggesting a role of TEAD1 in regulating HIF1A expression. Subsequently, ChIP experiments demonstrated that both the YAP1 and TEAD1 proteins can interact with the HIF1A promoter in HCT116 cells overexpressing HIF1A (Fig. 5l). These findings suggest that YAP1 interacts with TEAD1 to co-regulate HIF1A in CRC.

### YAP1 participates in the regulation of the biological functions of CRC cells through HIF1A

**Fig. 6:**
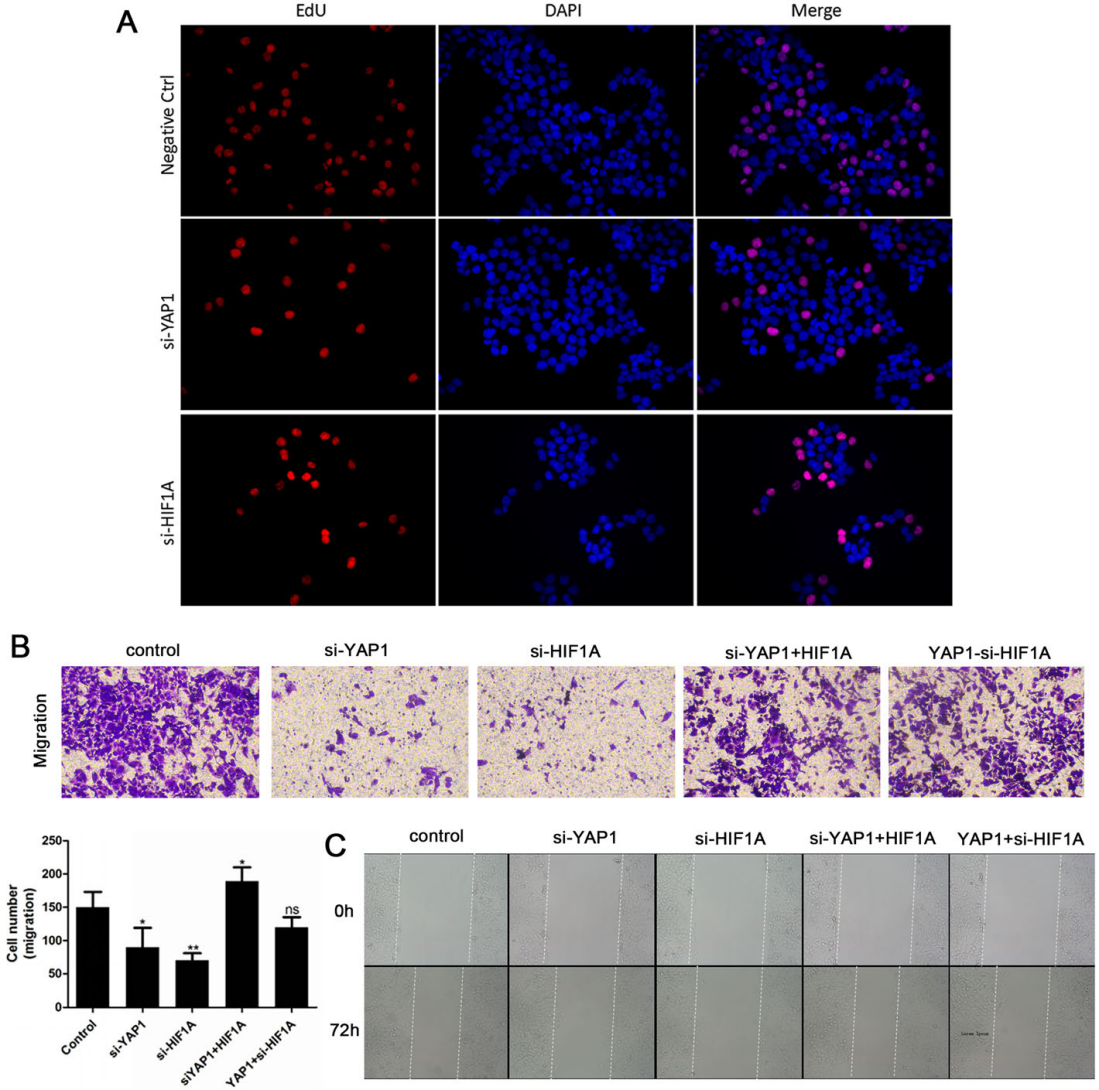
YAP1 modulates the biological functions of CRC cells through HIF1A. **a** The EdU cell proliferation assay in HCT116 cells transfected with vector, si-YAP1, or si-HIF1A. Transwell **b** and wound-healing **c** assays in HCT116 cells transfected with vector, si-YAP1, si-HIF1A, si-YAP1 + HIF1A, or YAP1 + si-HIF1A. *p < 0.05, **p < 0.01

To explore whether YAP1 serves its biological functions through HIF1A, a rescue experiment was designed using YAP1, si-YAP1, HIF1A and si-HIF1A. The EdU cell proliferation assay indicated that YAP1 or HIF1A knockdown inhibited the proliferation of HCT116 cells, compared with the negative control group (Fig. 6a). Transwell migration (Fig. 6b) and wound-healing (Fig. 6c) assays indicated that YAP1 or HIF1A knockdown attenuated the migration of HCT116 cells, whereas HIF1A overexpression partially reversed the effects of YAP1 knockdown, and YAP1 overexpression partially reversed the effects of HIF1A knockdown in the assays. Together, these findings support the notion that the regulatory roles of YAP1 in CRC biological functions are HIF1A-dependent.

### MiR-103a-3p affects glycolysis in CRC by regulating the hippo/YAP1/HIF1A axis

**Fig. 7:**
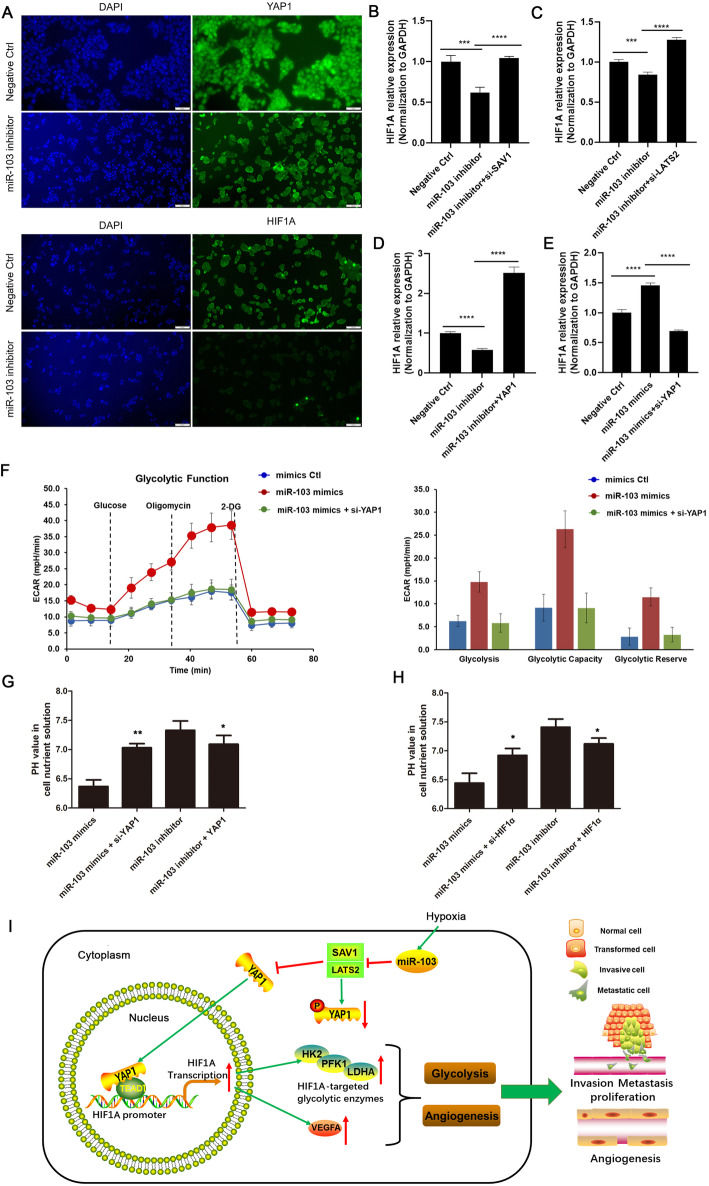
MiR-103a-3p affects glycolysis in CRC by regulating the Hippo/YAP1/HIF1A axis. **a** Immunofluorescence staining assay indicating that YAP1 and HIF1A localization was reduced in the nucleus and cytoplasm of HCT116 cells transfected with miR-103a-3p inhibitor under hypoxic conditions, respectively. **b-c** The regulation of miR-103a-3p-LATS2/SAV1 axis on HIF1A was determined in HCT116 cells transfected with miR-103a-3p inhibitor, miR-103a-3p inhibitor+si-SAV1, miR-103a-3p inhibitor+si-LATS2, or negative control by qRT-PCR. **d-e** The regulation of miR-103a-3p-YAP1 axis on HIF1A was determined in HCT116 and SW480 cells transfected with miR-103a-3p inhibitor, miR-103a-3p inhibitor+YAP1, or vector by qRT-PCR. **f** The ECAR and the variations of glycolysis, glycolysis capacity and glycolysis reserve of CRC cells were analyzed by Seahorse XFe 96 Extracellular Flux Analyzer. **g** The acidity of the cell culture medium was detected in SW480 and HCT116 cells transfected with miR-103a-3p mimics, miR-103a-3p mimics+si-HIF1A, miR-103a-3p inhibitor, or miR-103a-3p inhibitor+HIF1A. **h** The acidity of the cell culture medium was detected in SW480 and HCT116 cells transfected with miR-103a-3p mimics, miR-103a-3p mimics+si-YAP1, miR-103a-3p inhibitor, or miR-103a-3p inhibitor+YAP1. **i** Schematic representation of a model depicting the major molecular mechanisms of the miR-103a-3p-LATS2/SAV1-YAP1-HIF1A axis in CRC under hypoxic conditions

The above results demonstrated that miR-103a-3p directly bound to LATS2 and SAV1 and suppressed their activity, and YAP1/TEAD1 could activate the transcription of HIF1A. It is known that inactivated LATS2 and SAV1 inhibit YAP1 phosphorylation and promote YAP1 entry into the nucleus [24]. Therefore, we speculated that miR-103a-3p could promote YAP1 entry into the nucleus and affect the expression of HIF1A. Immunofluorescence assays confirmed that knockdown of miR-103a-3p markedly decreased the localization of YAP1 and HIF1A in the HCT116 cell nucleus and cytoplasm, respectively (Fig. 7a). Since the expression of HIF1A was regulated by miR-103a-3p targeting the LATS2/SAV1-YAP1 axis, whether miR-103a-3p completely or partially acts on the LATS2/SAV1-YAP1 axis remains to be determined. Therefore, we designed the corresponding rescue experiments. The knockdown of miR-103a-3p decreased the transcript level of HIF1A, whereas in the rescue experiment, the transcript level of HIF1A was reversed in HCT116 cells co-transfected with miR-103a-3p inhibitor and si-LATS2, si-SAV1 or YAP1 (Fig. 7b, c, d). Meanwhile, the overexpression miR-103a-3p increased the transcript level of HIF1A, whereas in the rescue experiment, the transcript level of HIF1A was reversed in SW480 cells co-transfected with miR-103a-3p mimics and si-YAP1(Fig. 7e). These results indicated that miR-103a-3p regulated the expression of HIF1A through the Hippo-YAP1 axis. Furthermore, to explore whether miR-103a-3p promotes tumour glycolysis through the YAP1-HIF1A axis, we designed the rescue experiments to detect changes in glycolytic function and changes in the acidity of the extracellular medium. The overexpression of miR-103a-3p enhanced the ECAR of SW480 cells, whereas in the rescue experiment, YAP1 knockdown restored the enhanced glycolytic metabolism of miR-103a-3p overexpression (Fig. 7f). In addition, overexpression or knockdown of miR-103a-3p increased or decreased the acidity of the cell nutrient solution in SW480 and HCT116 cells, respectively (Fig. 7g, h). In rescue experiments, YAP1 or HIF1A knockdown partially restored the effect of miR-103a-3p overexpression, and YAP1 or HIF1A overexpression partially restored the effect of miR-103a-3p knockdown (Fig. 7g, h). Taken together, these results demonstrated that miR-103a-3p promotes tumour glycolysis by regulating the Hippo/YAP1/HIF1A axis.

## Discussion

CRC is currently one of the most common malignancies diagnosed worldwide, and its morbidity and mortality have been on the rise in China for nearly a decade [1, 2]. As diagnostic and therapeutic strategies progress rapidly, especially the application of immunotherapy and molecular targeted biological therapy [38, 39], the overall survival rate of CRC has improved. Unfortunately, the overall prognosis of CRC remains poor, and new molecular diagnostics and therapeutic targets are urgently needed. In this study, we explored the effects of miR-103a-3p on CRC glycolysis and biological functions under hypoxic conditions by analysing clinical samples and performing experiments in vitro and in vivo.

The present research confirmed that miR-103a-3p was highly expressed in CRC and its high expression was closely associated with tumour metabolism and predicted poor prognosis. Further experiments showed that miR-103a-3p inhibits the activation of the Hippo pathway via inhibiting its targets LATS2 and SAV1. The process would increase the entry of YAP1 into the nucleus to upregulate the expression of HIF1A via binding to the transcriptional coactivator TEAD1. Furthermore, we demonstrated that HIF1A promoted the transcription of VEGFA and the glycolytic enzymes HK2, LDHA, and PFK1, ultimately promoting proliferation, invasion, migration and angiogenesis of CRC cells (Fig. 7i).

Intratumoural hypoxia plays a critical role in cancer progression, especially in cancer cell metabolism reprogramming [40]. As HIF1A is a hypoxia-stimulating factor, HIF1A-mediated regulation of a variety of genes and pathways, including angiogenesis and glycolysis, is crucial to cancer progression [41, 42]. In addition to being regulated by hypoxic levels, HIF1A is also affected by oncogenes and tumour suppressor genes. In this study, miR-103a-3p knockdown reduced the expression of HIF1A and the key molecules of glycolysis HK2, LDHA and PFK1. In addition, the regulatory effect of HIF1A on tumour metabolism and pro-angiogenic effects were demonstrated by bioinformatics analysis and cytology experiments. We hypothesized that miR-103a-3p regulates tumour metabolism and biological functions through HIF1A.

Previous evidence has demonstrated that hypoxia promotes the growth, glycolysis and stem cell potential of various tumours through the YAP/HIF1A signalling pathway [17, 43, 44]. It is well known that YAP1 is a transcriptional coactivator of the Hippo pathway that plays an oncogenic role in a variety of malignancies [37, 45]. Similarly, our research was focused on the exploration of how YAP1 regulated glycolysis in CRC. According to previous investigations and bioinformatics predictions, HIF1A could promote tumour glycolysis [46–48], and its promoter region might have a DNA binding motif for TEAD1, a transcription coactivator of YAP1. Furthermore, ChIP analysis proved that YAP1/TEAD1 could co-regulate the transcription of HIF1A and further promote tumour glycolysis. A rescue experiment was utilized to confirm that YAP1 serves its biological functions in CRC cells by regulating HIF1A. Subsequently, we studied the potential molecular mechanism by which miR-103a-3p is involved in this pathway.

Numerous miRNAs have been confirmed to be involved in the regulation of the Hippo pathway. For example, our previous research showed that miR-590-5p directly targets YAP1 and inhibits tumourigenesis in CRC cells [14]. In addition, a recent study showed that miR-103a-3p inhibits the Hippo pathway and activates YAP by directly targeting LATS2, ultimately promoting hepatoma cell metastasis and EMT [29]. LATS2 is the upstream regulator of YAP. Upon activation of the Hippo pathway, YAP is phosphorylated by activated LATS2 and subsequently confined to the cytoplasm or degraded [49]. In this study, LATS2 and SAV1 were confirmed as targets of miR-103a-3p in CRC cells and were inhibited by miR-103a-3p. Then, we performed the corresponding rescue experiments and indicated that miR-103a-3p promoted the expression of HIF1A and CRC glycolysis via targeting the LATS2/SAV1-YAP1-HIF1A axis.

## Conclusion

In summary, this project investigated the interactions between ncRNAs and YAP1 and their roles in the regulation of CRC glycolysis and tumour progression. MiR-103a-3p was found to be up-regulated in CRC and served as a tumour promoter. Our findings demonstrated that the miR-103a-3p-LATS2/SAV1-YAP1-HIF1A regulatory axis contributes to a better understanding of the molecular mechanisms of glycolysis in CRC, which would lay a theoretical foundation for molecular targeted therapy of CRC. Thus, miR-103a-3p could be regarded as a promising biomarker of CRC to improve individualized treatment for patients.

## Supplementary information

**Additional file 1 Table S1.** Association between miR-103a-3p and clinicopathological characteristics among 40 colorectal cancer patients. **Table S2.** Primer sequences for real-time PCR. **Table S3.** SiRNA sequences of related genes. **Figure S1.** The overall survival of glycolytic genes expression and relationship between miR-103a-3p and glycolytic genes in TCGA datasets. **Figure S2.** Correlation analysis of HIF1A and glycolytic genes expression levels in colon cancer, rectal cancer and CRC using GEPIA database. **Figure S3.** Correlation analysis of YAP1/TEAD1 and glycolysis-related gens expression levels in colon cancer, rectal cancer and CRC using GEPIA database.

## Data Availability

Data sharing not applicable to this article as no datasets were generated or analysed during the current study.

## Abbreviations

AJCC: American Joint Committee on Cancer
CRC: Colorectal cancer
ChIP: Chromatin immunoprecipitation
ECAR: Extracellular acidification rate
ESCC: Esophageal squamous cell carcinoma
EdU: 5-Ethynyl-2′-deoxyuridine assay
HIF: Hypoxia-inducible factor
H&E: Haematoxylin-eosin
IHC: Immunohistochemistry
MT: Mutant type
OS: Overall survival
UICC: International Union Against Cancer
WT: Wild type
YAP1: Yes-associated protein 1
