# Supplementary material for: MiR-103a-3p promotes tumour glycolysis in colorectal cancer via hippo/YAP1/HIF1A axis
Source: J Exp Clin Cancer Res. 2020 Nov 20;39:250. doi: 10.1186/s13046-020-01705-9 (PMC7678148; doi:10.1186/s13046-020-01705-9)
Supplement: Supplementary file 1 — Additional file 1 Table S1. Association between miR-103a-3p and clinicopathological characteristics among 40 colorectal cancer patients. Table S2. Primer sequences for real-time PCR. Table S3. SiRNA sequences of related genes. Figure S1. The overall survival of glycolytic genes expression and relationship between miR-103a-3p and glycolytic genes in TCGA datasets. Figure S2. Correlation analysis of HIF1A and glycolytic genes expression levels in colon cancer, rectal cancer and CRC using GEPIA database. Figure S3. Correlation analysis of YAP1/TEAD1 and glycolysis-related gens expression levels in colon cancer, rectal cancer and CRC using GEPIA database. [file 13046_2020_1705_MOESM1_ESM.zip › Supporting Information.docx]

Supporting Information

**Supplementary Figures**

**
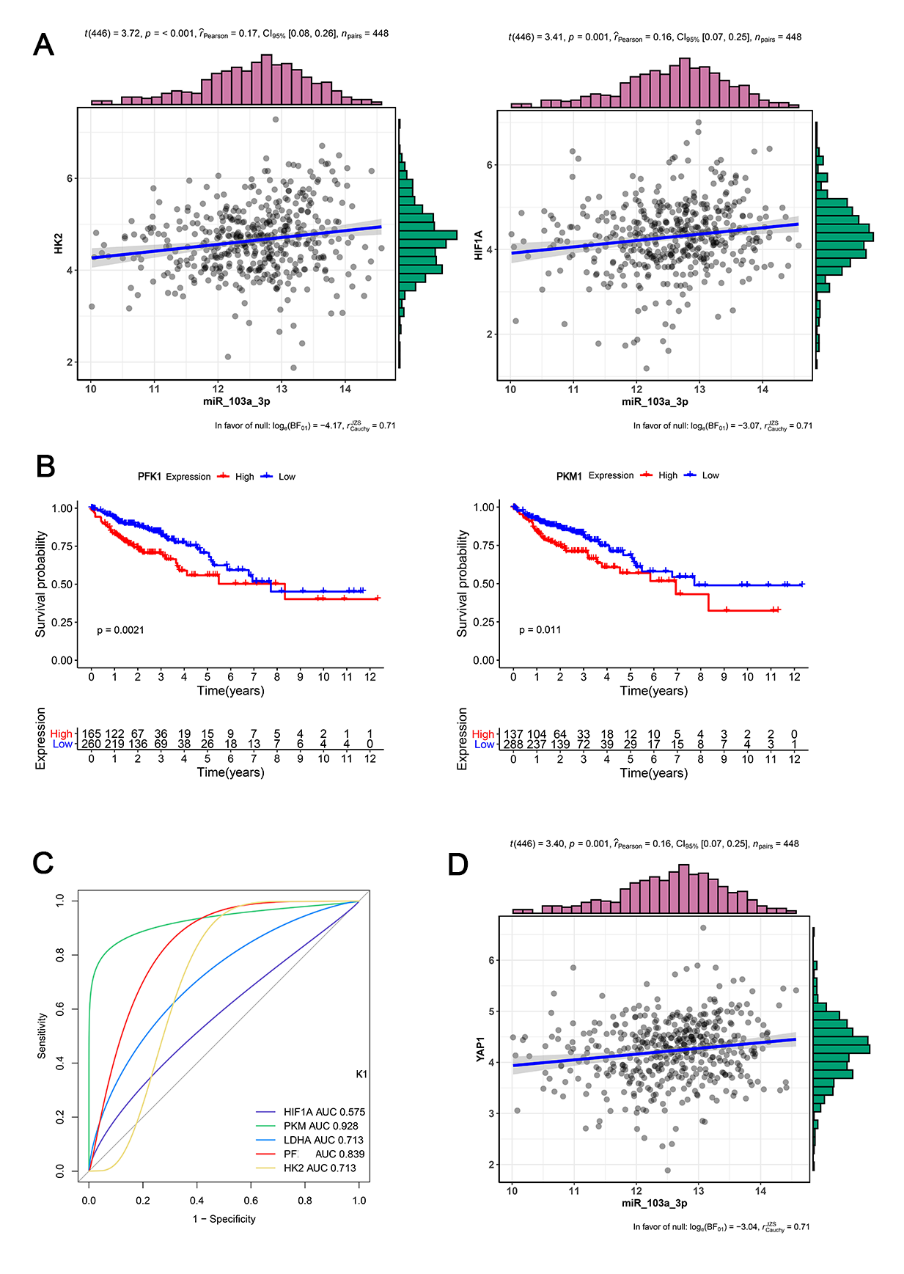
**

**Fig. S1** The overall survival of glycolytic genes expression and relationship between miR-103a-3p and glycolytic genes in TCGA datasets. (A) The expression correlation of miR-103a-3p with the glycolytic gene HK2 or HIF1A in the TCGA CRC datasets. (B) Kaplan-Meier curves showing the survival of CRC patients with high or low expression of PFK1 (cutoff value: 3.5) or PKM1 (cutoff value: 7.5). (C) ROC curves analysis of the sensitivity and specificity of HIF1A, PKM1, LDHA, PFK1 and HK2 in TCGA CRC datasets. (D) The expression correlation of miR-103a-3p with YAP1 in the TCGA CRC dataset.


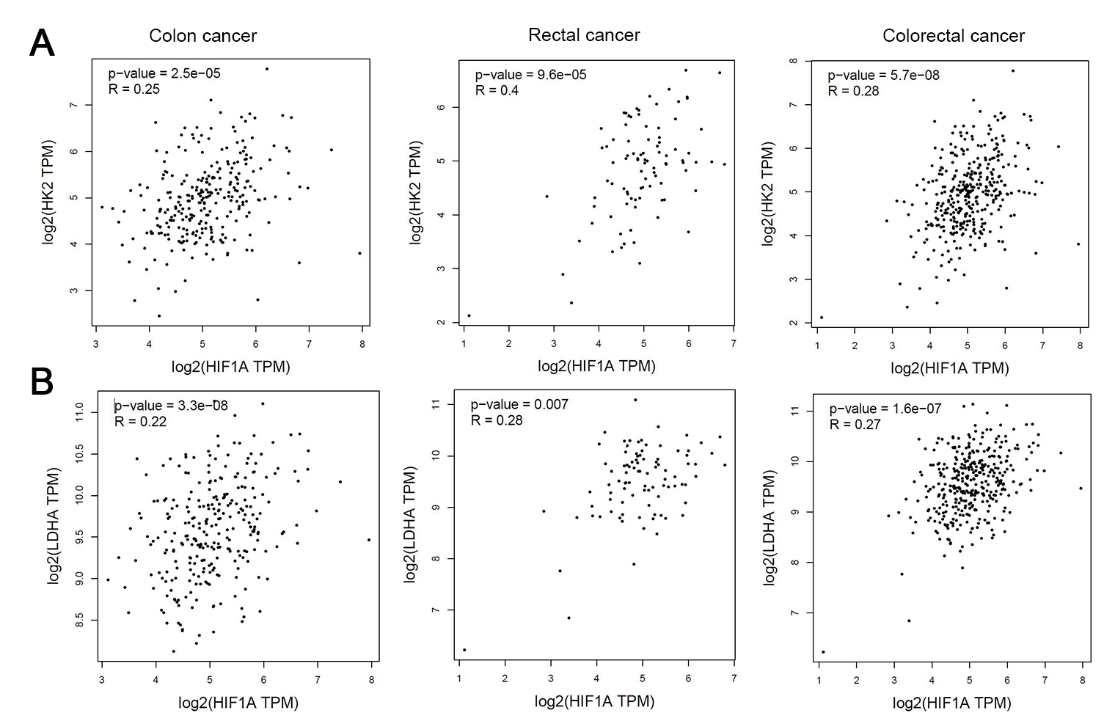


**Fig. S2** Correlation analysis of HIF1A and glycolytic genes expression levels in colon cancer, rectal cancer and CRC using GEPIA database. (A) The expression correlation of HIF1A with HK2 in colon cancer, rectal cancer and CRC tissues, respectively. (B) The expression correlation of HIF1A with LDHA in colon cancer, rectal cancer and CRC tissues, respectively.


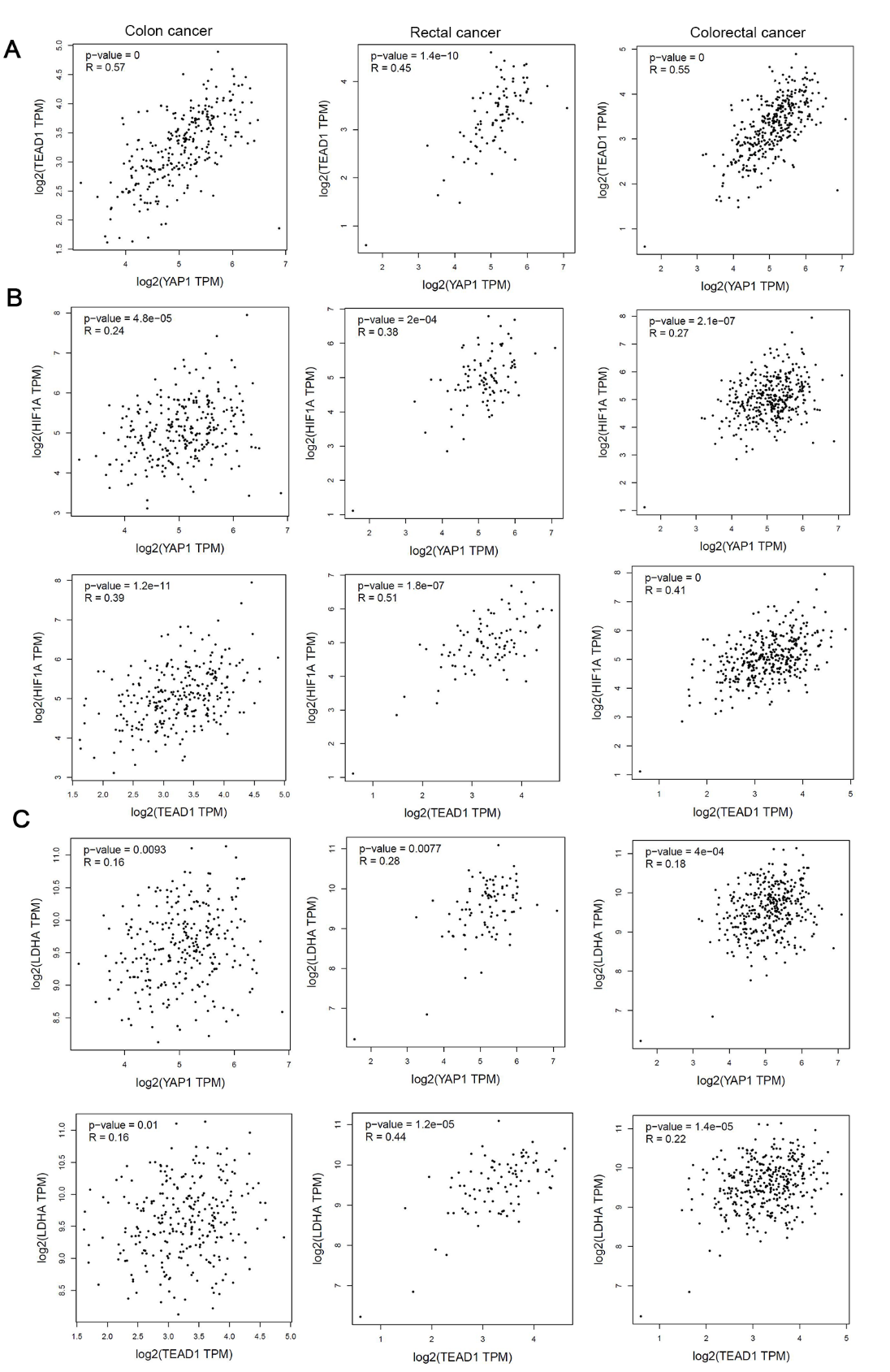


**Fig. S3** Correlation analysis of YAP1/TEAD1 and glycolysis-related gens expression levels in colon cancer, rectal cancer and CRC using GEPIA database. (A) The correlation of the YAP1 and TEAD1 levels in in colon cancer, rectal cancer and CRC samples. (B) The expression correlation of YAP1 or TEAD with HIF1A in colon cancer, rectal cancer and CRC tissues, respectively. (C) The expression correlation of YAP1 or TEAD1 with LDHA in colon cancer, rectal cancer and CRC tissues, respectively.

**Supplementary Tables**

**Table S1**

Association between miR-103a-3p and clinicopathological characteristics among 40 colorectal cancer patients

| Variables |  | miR-103 | | *X^2^* value | *P* value |
| --- | --- | --- | --- | --- | --- |
|  |  | Lower (n =18) | Higher (n =22) |  |  |
| Gender |  |  |  | 0.61 | 0.43 |
|  | Male | 12 | 12 |  |  |
|  | Female | 6 | 10 |  |  |
| Age |  |  |  | 0.22 | 0.64 |
|  | < 60 | 7 | 7 |  |  |
|  | ≥60 | 11 | 15 |  |  |
| Tumor size (cm) |  |  |  | 0.08 | 0.78 |
|  | <4 | 9 | 12 |  |  |
|  | ≥4 | 9 | 10 |  |  |
| Clinical stage |  |  |  | 0.17 | 0.68 |
|  | Ⅰ-Ⅱ | 6 | 6 |  |  |
|  | Ⅲ-Ⅳ | 12 | 16 |  |  |
| Distant metastasis |  |  |  | 3.74 | 0.05 |
|  | No | 15 | 12 |  |  |
|  | Yes | 3 | 10 |  |  |
| Venous invasion |  |  |  | 0.24 | 0.62 |
|  | No | 12 | 13 |  |  |
|  | Yes | 6 | 9 |  |  |
| Lymph node metastasis |  |  |  | 0.03 | 0.87 |
|  | No | 7 | 8 |  |  |
|  | Yes | 11 | 14 |  |  |

**Table S2**

Primer sequences for real-time PCR

| **Name** | **Forward primer (5’→3’)** |
| --- | --- |
| miR-103a-3p-F | CGCGAGCAGCATTGTACAGGG |
| miR-103a-3p-R | AGTGCAGGGTCCGAGGTATT |
| miR-103a-3p-RT | GTCGTATCCAGTGCAGGGTCCGAGGTATTCGCACTGGATACGACTCATAG |
| HK2-F | CCTCGGTTTCCCAACTCTGC |
| HK2-R | ACTGGTCAACCTTCTGCACT |
| LDHA-F | AGCTGTTCCACTTAAGGCCC |
| LDHA-R | TGGAACCAAAAGGAATCGGGA |
| PFK1-F | AATCTGCAAGAAAGCAGCGG |
| PFK1-R | GCAGCATTCATACCTTGGGC |
| PKM1-F | AGAACTTGTGCGAGCCTCAA |
| PKM1-R | GAGCAGACCTGCCAGACTC |
| HIF1A-F | TACTCAGCACTTTTAGATGCTGTT |
| HIF1A-R | ACGTTCAGAACTTATCCTACCAT |
| MST1-F | TATGGTTGGGCACCCTGTTC |
| MST1-R | CCCGTACCTTTGGTCTCACC |
| SAV1-F | CTCGTCGAGAAGGATGCTGT |
| SAV1-R | AGGCATAAGATTCCGAAGCAGA |
| LATS1-F | GAAACCAGGGAATGTGCAGC |
| LATS1-R | CGTTGCTAGGGTGAGCTTGA |
| LATS2-F | CGAATGTCCCACTTGGGTCTG |
| LATS2-R | TTGAAGATTATCACTCTCTCCAGGG |
| MOB1-F | AGGTTTGCAAAGGCTCGCAG |
| MOB1-R | TTAGAAGAGCGGCTGAAGAGG |
| YAP1-F | CCCTCGTTTTGCCATGAACC |
| YAP1-R | GTTGCTGCTGGTTGGAGTTG |
| TAZ-F | TGGACCAAGTACATGAACCACC |
| TAZ-R | CTGGTGATTGGACACGGTGA |
| TEAD1-F | CGAGACCCAGACTCGTACAAC |
| TEAD1-R | GAGGGCCCTTTCCAAACAGT |
| U6-F | CTCGCTTCGGCAGCACA |
| U6-R | AACGCTTCACGAATTTGCGT |
| GAPDH-F | AACGGATTTGGTCGTATTGG |
| GAPDH-R | TTGATTTTGGAGGGATCTCG |

**Table S3**

**siRNA sequences of related genes**

| si-LATS2 sense | GUUCGGACCUUAUCAGAAA |
| --- | --- |
| si-SAV1 sense | AAAUUCGGAUGACUCAACUCGUUCC |
| si-YAP1 sense | GGTGATACTATCAACCAAA |
| si-TEAD1 sense | GGATCAGACTGCAAAGGAT |
| si-HIF1A sense | GCTCCCAATGTCGGAGTTT |
