# Supplementary figures and images for: MiR-103a-3p promotes tumour glycolysis in colorectal cancer via hippo/YAP1/HIF1A axis
Source: J Exp Clin Cancer Res. 2020 Nov 20;39:250. doi: 10.1186/s13046-020-01705-9 (PMC7678148; doi:10.1186/s13046-020-01705-9)

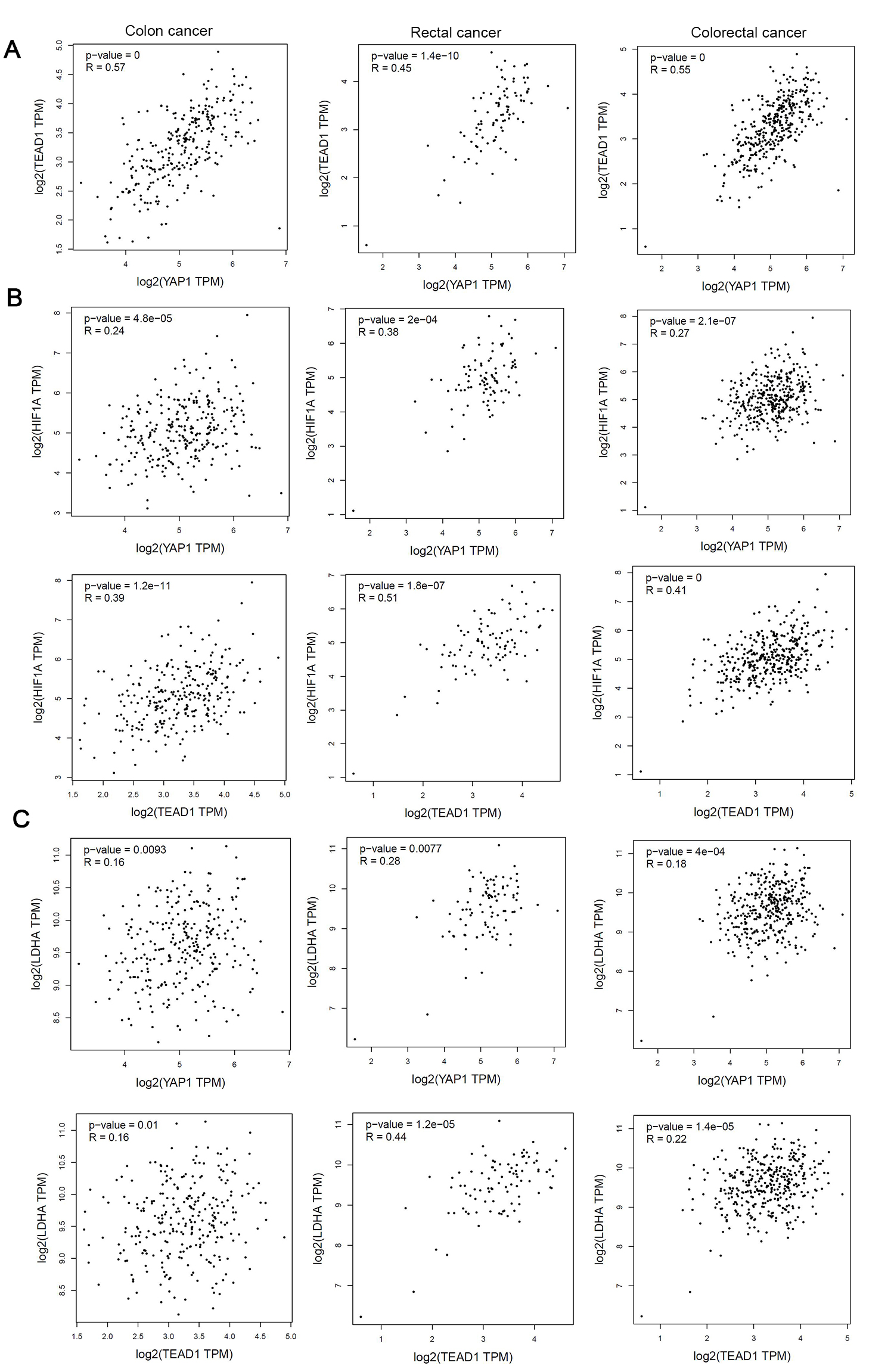

Supplement: Supplementary file 1 — Additional file 1 Table S1. Association between miR-103a-3p and clinicopathological characteristics among 40 colorectal cancer patients. Table S2. Primer sequences for real-time PCR. Table S3. SiRNA sequences of related genes. Figure S1. The overall survival of glycolytic genes expression and relationship between miR-103a-3p and glycolytic genes in TCGA datasets. Figure S2. Correlation analysis of HIF1A and glycolytic genes expression levels in colon cancer, rectal cancer and CRC using GEPIA database. Figure S3. Correlation analysis of YAP1/TEAD1 and glycolysis-related gens expression levels in colon cancer, rectal cancer and CRC using GEPIA database. [file 13046_2020_1705_MOESM1_ESM.zip › FIG-S3_ESM.jpg]

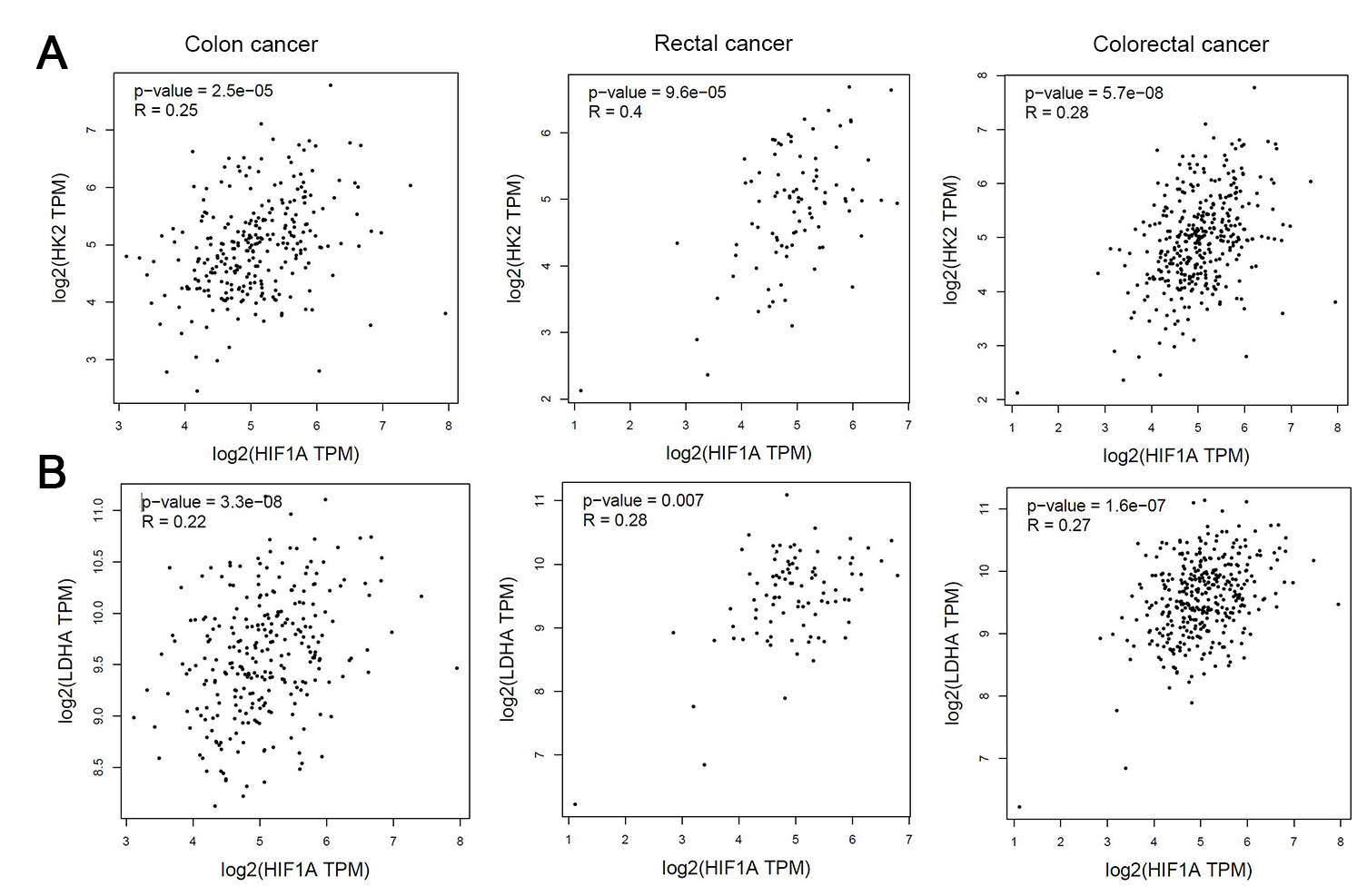

Supplement: Supplementary file 1 — Additional file 1 Table S1. Association between miR-103a-3p and clinicopathological characteristics among 40 colorectal cancer patients. Table S2. Primer sequences for real-time PCR. Table S3. SiRNA sequences of related genes. Figure S1. The overall survival of glycolytic genes expression and relationship between miR-103a-3p and glycolytic genes in TCGA datasets. Figure S2. Correlation analysis of HIF1A and glycolytic genes expression levels in colon cancer, rectal cancer and CRC using GEPIA database. Figure S3. Correlation analysis of YAP1/TEAD1 and glycolysis-related gens expression levels in colon cancer, rectal cancer and CRC using GEPIA database. [file 13046_2020_1705_MOESM1_ESM.zip › FIG-S2_ESM.jpg]
